# Temporary changes in STI & HIV testing & diagnoses across different phases of the COVID-19 pandemic, Chicago IL

**DOI:** 10.3389/frph.2023.1072700

**Published:** 2023-05-03

**Authors:** Maria Pyra, Tommy Schafer, Laura Rusie, Magda Houlberg, Hale M. Thompson, Anu Hazra

**Affiliations:** ^1^ Department of Data, Evaluation, & Epidemiology, Howard Brown Health, Chicago, IL, United States; ^2^ Institute for Sexual & Gender Minority Health & Wellbeing, Medical Social Sciences, Feinberg School of Medicine, Northwestern University, Chicago, IL, United States; ^3^ Division of Infectious Diseases, Department of Medicine, University of Chicago, Chicago, IL, United States

**Keywords:** HIV testing, STI testing, COVID- 19, MSM (men having sex with men), transgender

## Abstract

**Introduction:**

While the U.S. has seen a sustained rise in STI cases over the past decade, the impact of the COVID-19 on STIs and HIV is unclear.

**Methods:**

To examine the short- and medium-term impacts of COVID-19 and HIV and STI testing and diagnosis, we compared pre-pandemic trends to three periods of the pandemic: early- pandemic, March-May 2020; mid-pandemic June 2020-May 2021; and late-pandemic, June 2021-May 2022. We compared average number of monthly tests and diagnoses, overall and by gender, as well as the monthly change (slope) in testing and diagnoses.

**Results:**

We find that after decreases in average monthly STI and HIV testing and diagnoses during the early- and mid-pandemic, cases were largely back to pre-pandemic levels by the late-pandemic, with some variation by gender.

**Conclusion:**

Changes in testing and diagnoses varied by phase of the pandemic. Some key populations may require additional outreach efforts to attain pre-pandemic testing levels.

## 1. Introduction

The U.S continues to see rising cases of many STIs each year. The COVID-19 impacted access to testing as well as led to changes in sexual behaviors that could reduce transmission. The 2020 STD surveillance report shows that chlamydia, gonorrhea, and syphilis have all been increasing over the last decade; however, there were declines in the diagnosis of all three during the early months of the pandemic, leading to “uncertainty and difficulty in interpreting” the surveillance data from the pandemic (1). Preliminary data from 2021 suggest increasing cases for all three bacterial infections (2). Similar trends have been seen in Chicago, IL; chlamydia and syphilis cases have had an upward trend since at least 2003, while gonorrhea cases started increasing in 2015 (3).

Furthermore, the impacts of COVID-19 were not consistent but have been evolving since March 2020. Key changes during the early pandemic included changes in access to testing, shifts in public health and clinical staff to COVID-19 related projects, and changes in sexual behavior due to the emphasis on social distancing (4, 5). In addition, throughout the first year of the pandemic, there were also shortages of STI testing materials (6). However, access to testing, staffing, and sexual behaviors have not remained at the extreme lows seen in the early months of the pandemic. Interestingly, modeling suggests that duration of reduced sexual behavior compared to the duration of decreased testing access could lead to overall increases or decreases in HIV and STI cases, depending on the balance between the two (7). Based on the pandemic experience in Illinois, we defined pandemic time periods as: pre-pandemic, Jan 2017-Feb 2020; early-pandemic, March-May 2020, marked by the closing of schools and non-essential business, along with a shift to tele-medicine; mid-pandemic, June 2020-May 2021, described as gradually reopening, with intermittent restrictions re-imposed; and late-pandemic, June 2021-May 2022, noted for large scale vaccinations & masking restrictions removed (8).

In this paper, we seek to describe the short and medium-term changes in STI and HIV testing & diagnosis throughout multiple phases of the COVID-19 pandemic, specifically from March 2020 through May 2022, in order to better meet the current needs of STI & HIV testing.

## 2. Method

Data was extracted from electronic medical records (EMR) of patient visits (ages 18–90) with an HIV or bacterial STI (chlamydia, gonorrhea, or syphilis) test at a large sexual and gender minority focused federally qualified health center (FQHC) in Chicago, Illinois from January 2017 through May 2022. Bacterial infection screenings could be performed at any anatomical site (i.e., penile, vaginal, oropharyngeal, anal/rectal).

### 2.1. Statistical analysis

We examined four main outcomes: bacterial STI tests, bacterial STI diagnoses, HIV tests, and HIV diagnoses. HIV outcomes were restricted to those who were documented as HIV-negative at the time of testing. We first compared monthly averages for each outcome by pandemic phase, using *t*-tests (assuming unequal variance) and the pre-pandemic period as the reference; we repeated the comparisons of means, stratifying by self-reported gender (including cis men, cis women, trans men, transwomen, nonbinary, or other/unknown), our *a priori* primary predictor. To assess if the month to month changes for each outcome differed by pandemic phase, we then modeled the total monthly number of tests or diagnoses using linear regressions with time (month), pandemic period (as dummy variables), and time-pandemic period interactions, using an interrupted time-series approach (9). The slopes for each period were compared to the pre-pandemic period. We also graphed these changes over time, both overall and by gender; however due to small numbers we did not use gender in the interrupted time-series models. In the graphs, we also included the “counterfactual” of each model, which is the pre-pandemic slope continued over all time periods.

All analyses were conducted in SAS 9.4. The institution's IRB reviewed and deemed this study protocol exempt.

## 3. Results

Data were contributed by 72,443 unique patients across 257,412 visits, most of which occurred in the pre-pandemic phase (Table 1). This is a visit-level analysis, with most visits from young adults (45%), cis (cisgender) men (74%), and gay patients (of any gender) (50%); 41% identified as White, 27% as Black, and 22% as Hispanic/Latinx. People living with HIV prior to their study visit made up 21% of visits. There were few changes in demographics between the different pandemic phases; however, data collection did change with race/ethnicity and sexual orientation information being unreported for a larger proportion of patients starting in the early-pandemic.

**Table 1:** Demographics & testing across all visits with bacterial STI or HIV testing, by pandemic phase.

	Total	Pre-Pandemic (Jan ’17-Feb ’20)	Early Pandemic (Mar-May ’20)	Mid Pandemic (Jun ’20-May ’21)	Late Pandemic (Jun ’21-May ’22)
N, unique patients	72,443	55,278	6,129	22,410	26,481the
N, unique visits	257,412	159,277	6,835	40,005	51,295
Age					
18–24	16.8% (43,140)	18.2% (28,920)	15.4% (1,052)	15.1% (6,043)	13.9% (7,125)
25–34	44.5% (114,434)	43.8% (69,780)	44.7% (3,053)	45.2% (18,090)	45.8% (23,511)
35–44	20.1% (51,816)	19.3% (30,778)	21.1% (1,440)	21.0% (8,392)	21.9% (11,206)
45–54	9.9% (5,066)	11.3% (17,967)	10.6% (724)	10.4% (4,167)	9.9% (5,066)
55+	8.6% (4,387)	7.4% (11,832)	8.3% (566)	8.3% (3,313)	8.6% (4.387)
Gender					
Cis men	74.2% (190,926)	74.2% (118,181)	74.4% (5,086)	73.6% (29,431)	74.5% (38,228)
Cis women	14.4% (36,981)	13.8% (22,051)	14.3% (975)	15.1% (6,056)	15.4% (7,899)
Trans women	6.0% (15,381)	6.1% (9,663)	5.8% (399)	6.2% (2,484)	5.5% (2,835)
Trans men	3.1% (7,930)	3.4% (5,349)	2.8% (188)	2.8% (1,103)	2.5% (1,290)
Non-binary	1.1% (2,989	1.2% (1,948)	1.3% (88)	1.0% (396)	0.9% (466)
Other	1.3% (3,296)	1.3% (2,085)	1.5% (99)	1.3% (535)	1.1% (577)
Race/Ethnicity					
Black	27.4% (70,511)	26.0% (41,483)	30.1% (2,058)	30.6% (12,226)	28.7% (14,744)
White	40.6% (104,464)	43.7% (69,570)	35.9% (2,454)	34.6% (13,844)	36.3% (18,596)
Asian	5.2% (13,313)	5.4% (8,539)	4.9% (335)	4.9% (1,943)	4.9% (2,496)
Hispanic	21.6% (55,645)	21.5% (34,235)	22.3% (1,526)	22.1% (8.844)	21.5% (11,040)
Unknown	5.2% (13,479)	3.4% (5450)	6.8% (462)	7.9% (3,148)	8.6% (4,419)
Orientation					
Gay	50.4% (129,706)	55.3% (88,099)	50.7% (3,463)	45.3% (18,126)	39.0% (20,018)
Bisexual	8.5% (21,820)	9.4% (14,928)	9.6% (653)	7.4% (2,941)	6.4% (3,298)
Lesbian	0.9% (2,400)	1.1% (1,750)	1.1% (75)	0.7% (260)	0.6% (315)
Queer/Questioning	5.2% (13,443)	6.0% (9,485)	4.3% (293)	4.0% (1,606)	4.0% (2,059)
Straight	17.0% (43,672)	20.2% (32,240)	19.0% (1,298)	13.2% (5,296)	9.4% (4,838)
Other/Unknown	18.9% (46,371)	8.0% (12,775)	15.4% (1,053)	29.4% (11,776)	40.5% (20,767)
Living with HIV	21.2% (54,435)	20.6% (32,733)	23.6% (1,610)	23.0% (9,192)	21.3% (10,900)

### 3.1. Bacterial STIs

Before the COVID-19 pandemic, the average number of monthly bacterial STI tests (chlamydia, gonorrhea, or syphilis) was 3,807 (SD 825; Table 2); this decreased in the early- and mid-pandemic phases, although it was only statistically significantly lower in the mid pandemic. Monthly tests returned to a similar average in the late-pandemic (4,103 [SD 254]). Positive bacterial STI tests followed a similar pattern, with 618 (SD 118) average monthly diagnoses in the pre-pandemic phase, decreased positive results in the early- and mid-pandemic, with the latter statistically significant (523 [SD 70]), and a non-significant increase to 622 (SD 55) in the late-pandemic phase. When stratified by gender, patterns were overall similar, though there were some differences in which timepoints met statistical significance. Of note, cis women had significantly higher monthly tests in the late-pandemic compared to pre-pandemic, while trans men and non-binary patients had significantly lower monthly tests. In terms of positive tests by gender, trans women had a significantly lower average monthly diagnoses in the early-pandemic compared to the pre-pandemic, and trans men and non-binary patients had significantly lower diagnoses in the late-pandemic.

**Table 2:** Average monthly testing & diagnosis, Any bacterial STI or HIV, by gender*.

Any Bacterial STI	Pre-Pandemic (Jan ’17-Feb ’20)	Early Pandemic (Mar-May ’20)	Mid Pandemic (Jun ’20-May ’21)	Late Pandemic (Jun ’21-May ’22)
Average Monthly Tests (SD), Total	3,807 (825)	2,168 (1,026)	**3,186** **(397)**	4,103 (254)
Cis men	2,858 (572)	1,622 (763	**2,350** **(283)**	3,064 (185)
Cis women	520 (164)	307 (140)	480 (77)	**632** **(49)**
Trans women	218 (45)	123 (60)	194 (31)	222 (24)
Trans men	115 (29)	59 (29)	**88** **(13)**	**102** **(15)**
Non-binary	46 (15)	26 (21)	**32** **(7)**	**37** **(9)**
Other	49 (14)	31 (16)	**43** **(7)**	46 (9)
Average Monthly Diagnoses (SD)	618 (118)	406 (141)	**523** **(70)**	622 (55)
Cis men	524 (93)	341 (11)	**432** **(59)**	530 (48)
Cis women	45 (18)	34 (7, 62)	47 (9)	48 (6)
Trans women	26 (7)	**14** **(3)**	27 (6)	27 (5)
Trans men	9 (5)	6 (5)	7 (2)	**6** **(3)**
Non-binary	7 (3)	5 (3)	**5** **(3)**	**4** **(2)**
Other	7 (4)	6 (4)	6 (2)	7 (2)
HIV	Pre-Pandemic (Jan ’17-Feb ’20)	Early Pandemic (Mar-May ’20)	Mid Pandemic (Jun ’20-May ’21)	Late Pandemic (Jun ’21-May ’22)
Average Monthly Tests (SD)	2,743 (511)	1,372 (724)	**2,043** **(242)**	2,716 (187)
Cis men	1,996 (351)	996 (513)	**1,505** **(185)**	1,997 (128)
Cis women	413 (110)	212 (106)	**325** **(45)**	454 (38)
Trans women	161 (28)	77 (47)	**112** **(20)**	**137** **(17)**
Trans men	95 (15)	**40** **(22)**	**51** **(8)**	**72** **(10)**
Non-binary	40 (13)	23 (22)	**24** **(5)**	**27** **(8)**
Other	38 (10)	23 (15)	**27** **(5)**	**30** **(6)**
Average Monthly Diagnoses (SD)	17 (4)	**9** **(2)**	15 (5)	**12** **(6)**
Cis men	14 (4)	**7** **(1)**	13 (4)	**9** **(5)**
Cis women	0.5 (0.7)	0.7 (0.6)	0.3 (0.5)	0.9 (1)
Trans women	1 (1)	0.3 (0.6)	1 (1.0)	1 (1)
Trans men	0.2 (0.4)	**0** **(0)**	0.1 (0, 0.3)	0.1 (0.3)
Non-binary	0.1 (0.3)	**0** **(0)**	**0** **(0)**	0.3 (0.9)
Other	0.5 (0.8)	0.3 (0.6)	0.3 (0.5)	**0** **(0)**

* Means tested by *T*-test, unequal variance. Bold indicated *p *< 0.05, compared to pre-pandemic period.

**Figure 1:**
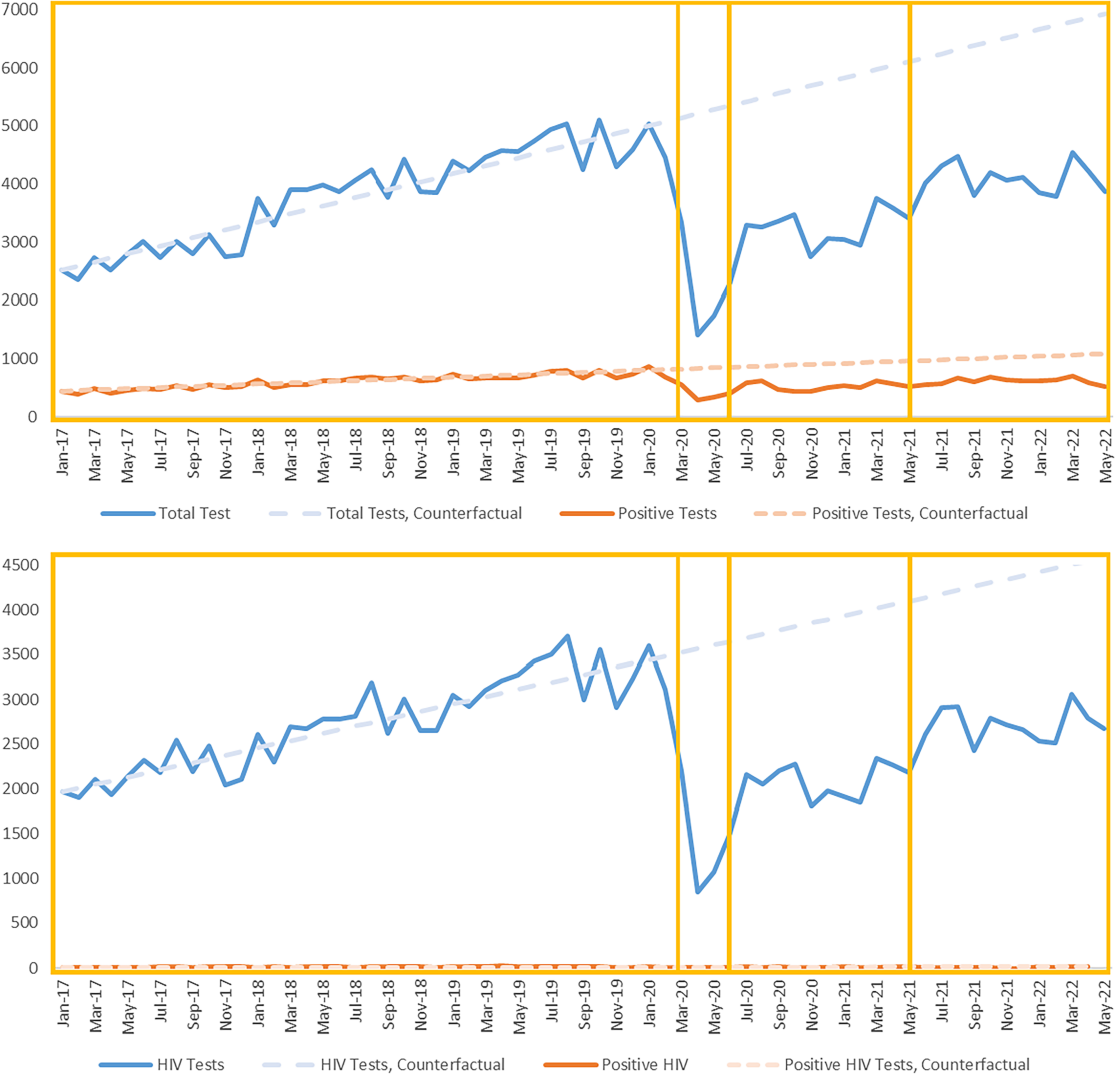
Total tests & positive tests over time, Any bacterial STI or HIV, by pandemic phase. The aggregate number of monthly tests (upper line) and positive tests (lower line) for any bacterial STI (upper pane) or HIV (lower pane) are shown as line graphs. The boxes delineate the Pre-, Early-, Mid-, and Late-Pandemic Phases. The dotted lines, marked counterfactual, are continuations of the pre-pandemic slope from each corresponding model.

As Figure 1 and Table 3 show, the slopes in both testing and positive tests differed visually by each pandemic phase. Pre-pandemic there was an increasing slope in testing and positive tests (69 and 10, respectively); this rapidly and significantly became a decreasing slope during the brief earl- pandemic phase (Table 3). Slopes began increasing again in the mid-pandemic phase, with a slight decline in the late-pandemic—although neither of these two timepoints were significantly different from the pre-pandemic phase. The percent of tests positive (positivity rate) was 16.3% in the pre-, 18.7% in the early-,16.4% in the mid-, and 15.2% in the late-pandemic periods.

**Table 3:** Changes in testing & diagnosis slopes, Any bacterial STI or HIV*.

Any Bacterial STI	Pre-Pandemic (Jan ’17-Feb ’20)	Early Pandemic (Mar-May ’20)	Mid Pandemic (Jun ’20-May ’21)	Late Pandemic (Jun ’21-May ’22)
Monthly Change in Testing (95% CI)	69 (60, 78)	**−795** **(−1,229, −362)**	54 (3, 105)	**−**11 (**−**63, 40)
Monthly Change in Diagnoses (95% CI)	10 (8, 11)	**−107** **(−182, −32)**	6 (**−**3, 14)	0.03 (**−**9, 9)
HIV	Pre-Pandemic (Jan ’17-Feb ’20)	Early Pandemic (Mar-May ’20)	Mid Pandemic (Jun ’20-May ’21)	Late Pandemic (Jun ’21-May ’22)
Monthly Change in Testing (95% CI)	41 (3, 35)	**−564** **(−872, −256)**	27 (**−**9, 64)	1 (**−**36, 37)
Monthly Change in Diagnoses (95% CI)	0.08 (**−**0.05, 0.2)	0.5 (**−**6, 7)	0.2 (**−**0.6, 0.9)	0.05 (**−**0.2, 1.2)

* Monthly changes from slope term (time + time*phase interaction) for each pandemic period in linear regression models. Bold indicated *p *< 0.05, compared to pre-pandemic period.

### 3.2. HIV

Prior to the COVID-19 pandemic, there were an average of 2,743 (SD 511) HIV tests and 17 (SD 4) HIV diagnoses each month. These both declined in the early-pandemic phase, with a significant drop in HIV diagnoses (9 [SD 2]). This decline continued in the mid-pandemic phase, with HIV tests significantly lower than the pre-pandemic period. While HIV tests have returned to levels similar to the pre-pandemic, HIV diagnoses remain slightly lower, at 12 (SD 6) per month. When examining these patterns by gender, cis men and cis women have the same overall pattern, but all other genders showed significantly lower HIV testing still in the late-pandemic compared to pre-pandemic. However, cis men were the only group to have significantly lower HIV diagnoses in the late-pandemic phase.

In terms of changes in slope (Figure 1; Table 3), HIV testing and diagnoses had been increasing month over month prior to the pandemic (slopes of 41 and 0.08 respectively; there was a large decline in testing during the early-pandemic that was significant (Table 3). Testing and diagnoses slopes began to increase again in the mid-pandemic and have slightly declined or stayed flat in the late-pandemic phase. Positivity rates were 0.60%, 0.63%, 0.72%, and 0.38% from early- to late-pandemic phases.

## 4. Discussion

In this analysis, we found that the average monthly tests and diagnoses for bacterial STIs was lower but statistically significant during the early-pandemic. This decrease continued and was statistically significant (likely due to the larger sample size) through the mid-pandemic phase. However, by the late-pandemic, testing and diagnoses had returned to pre-pandemic levels or higher, with some variations by gender. There was a similar pattern for HIV, although HIV diagnoses were significantly lower in the early- and late-pandemic phases. For all testing and diagnoses, there was a sharp decline in the slope (that is, the month to month change) during the early-pandemic compared to pre-pandemic.

It is difficult to assess whether changes during the pandemic were due to behavior or access; in actuality the causes were likely multifactorial. The CDC found a decrease both in HIV testing and diagnosis from February through December 2020, compared to the year prior (10). Similar to our results, most of this difference occurred early in the pandemic, with testing returning but not quite research previous levels by the fall (10). Multiple studies found rapid decline in bacterial STI diagnoses and testing during early pandemic, with some interesting variations by race & ethnicity (11–13). Two other studies found lower testing and diagnoses for most STIs & HIV during the early-pandemic period, but a subsequent increase in diagnoses in the mid-pandemic (14, 15). A study in a pediatric primary care network found that the first 8 months of the pandemic (compared to the previous year) showed fewer STI tests but a similar number of cases (16). Finally, study in Washington state found a larger decrease in asymptomatic compared to symptomatic cases during the early pandemic, suggesting declines are due more to limited screening; however, this decline could have been related to either access or test-seeking behaviors (13).

In terms of behavior, U.S. survey of gay men at early (April-May 2020) and mid (November 2020-January 2021) pandemic timepoints found little evidence that number of sexual partners changed from pre-pandemic times (17). However 32% and 19% (respectively at early- and mid-timepoints) reported the pandemic made HIV testing difficult, while 29% and 18% reported the same for STI testing (17). Reported PrEP use was similar at both timepoints (27% & 25%) (17), indicating either that sexual activity was consistent or simply that PrEP users were comfortable with their PrEP routine even in the absence of sex. However, other studies have found changes in sexual practices, particularly in the early-pandemic period (18, 19).

This study has certain limitations. Due to changes in data collection during the pandemic, we are not able to compare trends by race and/or ethnicity. We cannot account for changes in outreach or programming at different clinical sites that may have impacted testing among certain populations; we used all testing results, including STI walk-in services and primary care, which could have been differentially impacted. We did not collect data on behavior and therefore cannot separate out the effects of testing vs. sexual transmission. For multiple reasons, patients may not have chosen to report their actual gender, leading to possible misclassification. These data are from a sexual and gender minority focused health systems and the pandemic restrictions experienced in Chicago, IL likely differ from other areas of the nation; therefore these results are likely not generalizable to all settings.

In conclusion, we extend previous findings related to the early- and mid-pandemic phases. We find that HIV and STI testing and diagnoses had changed dramatically over different phases of the COVID-19 pandemic. However, by the late-pandemic phases—even including the Omicron wave—testing rates had largely returned to pre-pandemic levels, although they would likely have been higher in the pre-pandemic trajectories had continued uninterrupted. This pattern may help public health officials make decisions around resources during the acute phase of future public health emergencies. Testing and diagnoses appear to remain lower in some transgender and non-binary populations; in addition to improved outreach and communication with members of these communities, more research around both data collection and the impact of anti-trans stigma on disclosure of gender identity and sexual risk behaviors may be helpful to re-establish care and ensure latent infections are not being missed. Finally, using implementation science to understand how these clinics were able to restore high levels of testing would be a useful area of future research.

## Data Availability

The data analyzed in this study is subject to the following licenses/restrictions: De-identified data are available by request. Requests to access these datasets should be directed to Maria Pyra, maria.pyra@northwestern.edu.
